# Pathogens at the livestock-wildlife interface in Western Alberta: does transmission route matter?

**DOI:** 10.1186/1297-9716-45-18

**Published:** 2014-02-12

**Authors:** Mathieu Pruvot, Susan Kutz, Frank van der Meer, Marco Musiani, Herman W Barkema, Karin Orsel

**Affiliations:** 1 Faculty of Veterinary Medicine, University of Calgary, 3330 Hospital Drive, Calgary, NW, Alberta T2N 4N1, Canada; 2 Faculty of Environmental Design, University of Calgary, 2500 University Drive, NW, Calgary, AB T2N 1N4, Canada

## Abstract

In southwestern Alberta, interactions between beef cattle and free-ranging elk (*Cervus elaphus*) may provide opportunities for pathogen transmission. To assess the importance of the transmission route on the potential for interspecies transmission, we conducted a cross-sectional study on four endemic livestock pathogens with three different transmission routes: Bovine Viral Diarrhea Virus and Bovine Herpesvirus 1 (predominantly direct transmission), *Mycobacterium avium* subsp. *paratuberculosis* (MAP) (indirect fecal-oral transmission), *Neospora caninum* (indirect transmission with definitive host). We assessed the occurrence of these pathogens in 28 cow-calf operations exposed or non-exposed to elk, and in 10 elk herds exposed or not to cattle. We characterized the effect of species commingling as a risk factor of pathogen exposure and documented the perceived risk of pathogen transmission at this wildlife-livestock interface in the rural community. Herpesviruses found in elk were elk-specific gamma-herpesviruses unrelated to cattle viruses. Pestivirus exposure in elk could not be ascertained to be of livestock origin. Evidence of MAP circulation was found in both elk and cattle, but there was no statistical effect of the species commingling. Finally, *N. caninum* was more frequently detected in elk exposed to cattle and this association was still significant after adjustment for herd and sampling year clustering, and individual elk age and sex. Only indirectly transmitted pathogens co-occurred in cattle and elk, indicating the potential importance of the transmission route in assessing the risk of pathogen transmission in multi-species grazing systems.

## Introduction

The foothills of the Canadian Rocky Mountains in southwestern Alberta, Canada are extensively grazed by cow-calf herds and free-ranging North American elk (*Cervus elaphus*). With similar grazing patterns [1] and diet [2-4], cattle and elk have multiple opportunities for interspecies pathogen transmission, as observed with *Brucella abortus* and *Mycobacterium bovis* elsewhere in North America [5-8]. In multi-host systems, wildlife or domestic species may act as reservoir or spillover hosts [9-12], or be part of the maintenance community [13], for a number of livestock pathogens.

Among factors influencing the potential for interspecies pathogen transmission (related to pathogen biology, wildlife behavior and ecology, and livestock management), the transmission route of the pathogen is of particular interest, and has previously been discussed as a factor related to the emergence of zoonotic pathogens [14-16]. However, there is still much to learn about the relevance of transmission pathways in multi-host systems in general [17]. Because pathogens with different transmission pathways may require very different prevention and control strategies, understanding the relative importance of transmission routes in multi-host systems is essential [17].

The influence of the transmission route on the potential for inter-species transmission can be either intrinsic or extrinsic to the pathogen. Intrinsic factors are related to the biological ability of a pathogen to infect different host species (host specificity, environmental persistence) and the evolutionary relationships between transmission route, taxonomic class, and host-range. Some authors have indeed suggested that there may be evolutionary advantages for indirectly transmitted pathogens to be generalists and therefore have a wider host range [18]. It has also been previously observed that indirectly transmitted and vector-borne pathogens are more likely to be zoonotic (a particular case of multi-host) pathogens [19]. Nevertheless, multi-species pathogens can be found with any type of transmission route and in any taxonomic group.

Extrinsic factors influencing inter-species transmission are related to the spatio-temporal constraints imposed by a given transmission route. Transmission of a pathogen between two species is only possible if the inter-species contact patterns meet the conditions required for the pathogen’s transmission route. The spatio-temporal contact structure of two species is influenced by behavioral, ecological and management factors. But with a given interaction pattern, it is the transmission route of a pathogen that will likely influence the number of transmission events occurring between the two species. Following this hypothesis, we would expect indirectly transmitted pathogens to be more easily shared between species than directly transmitted ones, because they do not require a strict temporal and/or spatial sympatry.

In this study, we selected different cattle production-limiting diseases [20] as models for different transmission routes at the interface between beef cattle and elk. Bovine Viral Diarrhea Virus (BVDV) and Bovine Herpesvirus 1 (BHV1) were chosen as examples of pathogens transmitted by direct contact. *Mycobacterium avium* subspecies *paratuberculosis* (MAP) would most likely be indirectly transmitted between cattle and elk through environmental fecal contamination. Finally, *Neospora caninum* has a more complex lifecycle with vertical transmission from dam to calf and horizontal transmission involving carnivores as definitive hosts. Presence of these four pathogens was previously reported in beef cattle in the study area [21-23] and there is substantial evidence that elk (or closely related red deer [*Cervus elaphus*]) are susceptible to these same pathogens [24-31].

In this study, we determined the presence of, or evidence of exposure to, these four pathogens in cattle and elk. We then characterized the importance of inter-species *commingling* (broadly defined throughout this manuscript as *habitat overlap*) as a risk factor for the circulation of these pathogens in both species. Finally, human dimension considerations in wildlife health studies are important to get a better understanding of people’s perception of disease risk and monitor their attitude toward wildlife management in general [32]. We therefore interviewed ranchers to document their perception of the risk of inter-species pathogen transmission.

## Materials and methods

### Elk herd description and sampling

**Figure 1:**
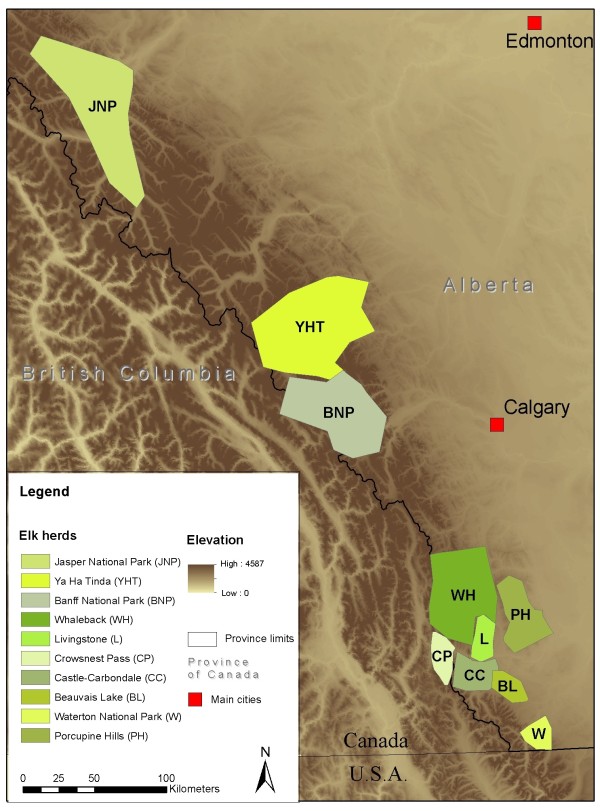
**Schematic winter home ranges of ten elk herds in Alberta, Canada.** Map indicating the approximate extent and location of ten elk herds in western Alberta. Main Alberta cities and administrative borders are represented. The map background represents the elevation.

Ten elk herds from the foothills of central and southern Alberta were classified to 2 groups: five with a high level of interaction with cattle (exposed elk), and five with limited to no contact with cattle (non-exposed elk) (Figure 1). Exposed elk were from herds whose winter home-range completely overlapped private land used for cattle grazing, based on telemetry data from collared elk and provincial ungulate winter surveys. The home-range of non-exposed herds did not significantly overlap private land and were mainly located in national parks or other protected public land. Herd names, sizes and additional information relevant to their classification as exposed or non-exposed are detailed in Table 1. Two hundred ninety-nine serum samples and individual data were collected from captured elk from these 10 herds, 86 fecal samples were collected at capture from a subset of the elk from 7 herds (Table 1).

**Table 1:** Description of elk samples obtained from ten herds across Western Alberta

**Herd (abbreviation)**	**Exposure to cattle**	**Estimated herd size**	**Captured elk**			**Additional fecal sample collection**	**Herd description literature**
			**Project**	**Serum samples**	**Fecal samples at capture**		
Beauvais lake (BL)	Exposed	150-250^e^	Montane Elk Research Program	9	8	69	[33-36]
Castle-carbondale (CC)		500-700^e^	Montane Elk Research Program	72	34	80	
Livingstone (L)		340^e^	Montane Elk Research Program	16	12	74	
Porcupine hills (PH)		450-700^e^	Montane Elk Research Program	8	3	69	
Whaleback (WH)		700-1000^e^	Montane Elk Research Program	30	12	93	
Waterton (W)^a^	Non-exposed	900^e^	Montane Elk Research Program	16	10	64	
Crowsnest pass (CP)^b^		200^e^	Montane Elk Research Program	17	7	38	
Jasper National Park (JNP)^c^		1300^f^	Parks Canada	31	0	55	[37]
Banff National Park (BNP)^c^		215^f^	Parks Canada	20	0	NA	[38]
Yaha Tinda (YHT)^d^		1000^f^	University of Alberta - University of Montana	80	0	70	[39]

^a^The elk herd only partially spread outside the park boundaries during calving season.

^b^Cattle are only present for summer public grazing.

^c^JNP and BNP are resident herds of the national parks and have no interaction with cattle.

^d^Although the herd has recently been observed to extend its home range eastward toward ranched areas (Merrill, personal communication), this herd has had very limited contact with cattle over the last several years.

^e^Source: Alberta Conservation Association Winter Survey 2001-2002 and 2006.

^f^Source: Parks Canada.

Additional fresh fecal samples were collected from all herds except Banff National Park (BNP) in the winter of 2010. To collect fresh samples from distinct individuals from the different sub-groups of the 9 herds, we took into account the ecology and behavior of elk and applied the following sampling strategy. An observation phase aimed at identifying sub-groups of a herd, and observing their connections, dynamics, and movement patterns. The known home range of each herd was searched with the help of information from local observers, GPS collar data, and localization by radio frequency triangulation. Since sub-group structure can change quickly, sub-groups in close proximity and highly connected were sampled the same day and never revisited to avoid the re-sampling of individuals. Sampling pellet piles left in resting areas significantly reduced the risk of sampling the same individual twice (distinct track in snow). If individuals were far apart, the observer would identify landmarks to collect pellets from distinct individuals. On site, only the top of structured fresh pellet piles (based on consistency and color) were sampled to avoid cross-contamination between samples and with the soil. A total of 616 additional fecal samples were collected in this manner from the 9 elk herds between March and May 2010 (Table 1). Fecal samples from BNP were opportunistically obtained from Parks Canada wildlife officers during collaring operations, culling of nuisance animals, or road kill removal.

### Cattle herd selection and sampling

We recruited 30 ranches, 15 exposed to elk and 15 having no contact with elk. Our inclusion criteria for ranch selection were cow-calf operations larger than 100 adult cattle, situated in the municipal districts of Pincher Creek, Crowsnest Pass, Willow Creek or Cardston. The classification regarding the exposure to elk was performed using: delimitation of elk home range, elk telemetry data, and discussion with local ranchers, veterinarians, and biologists.

Initial groups of 24 exposed and 30 unexposed ranches were identified by local veterinarians, thorough exploration of the study area, and preliminary ranch visits. Four and six of these herds, respectively, did not meet the eligibility criteria. In each group, 15 ranches agreed to be part of the study.

Thirty cows from each ranch were randomly selected among cows at their second calving (> 2 years old) and older by systematic sampling (1 sample every N/30 cows, with N the total herd size meeting this criteria, the first cow being randomly picked), allowing for the detection of a proportion of positive individuals of 10% with 95% confidence. MAP has a long incubation period and sampling older cows increases the probability of detection in the herd. A fecal sample was collected from the rectum and a blood sample from the coccygeal vein on each cow. Individual data included: animal ID, sex, age, breed, origin (born on ranch or purchased), pregnancy status, parity, and known past health history. To increase the probability of detecting BVDV circulation or presence of persistently infected animals in the different herds, we additionally collected serum samples from weak, sick or poor growing calves [40].

### Ranch management and risk perception data collection

A self-administered questionnaire and a follow-up one-on-one interview collected information on ranch characteristics, including herd size, average number of cows per bull, conception rate, weaning rate, and calf morbidity. This allowed comparison of the surveyed ranches with published benchmark data for cow-calf operation characteristics in Alberta [41]. We also documented the perception of interspecies disease transmission risk by the interviewed ranchers. In particular, the perceived likelihood of cattle-elk disease transmission (with no reference to a specific pathogen) and the level of agreement with the statement “Elk diseases should be monitored” were measured on a continuous scales (*very unlikely* to *very likely*, and *strongly disagree* to *strongly agree*, respectively). These continuous scales were transformed into discrete scores between 0 and 4 in the data analysis.

Questionnaire and interview documents were internally and externally reviewed, tested in the field prior to the study start, and approved by the University of Calgary Conjoint Faculties Research Ethics Board (file no. 6598). Questionnaire and interview materials are available upon request.

### Laboratory procedures

#### Pestivirus

We used the Pourquier® ELISA BVD/MD/BD P80 Antibodies Kit (competitive ELISA; Institut Pourquier, Montpellier, France), according to manufacturer instructions on both cattle and elk sera. This test detects antibodies against BVDV and Border Disease Virus (BDV), and possibly other pestiviruses since the P80 protein (NS3) is highly conserved among pestiviruses [42]. BVDV-positive and -negative elk sera were provided by the Lethbridge Animal Health Laboratory [27] and included in each plate.

Virus cross-neutralization was conducted at the National Animal Disease Center, USDA as described previously [43] on all seropositive elk samples, 10 seronegative individuals from matching herds, and 4 experimentally infected elk: 2 with the strain BVDV1 Singer and 2 with the strain BVDV2 24514. Comparison of neutralizing titers were made between 6 known pestivirus strains: CoosBay5c (BDV), BVDV2-296c, BVDV1a-Singer, BVDV1b-TGAC, Pronghorn pestivirus [44] and “HoBi”-like pestivirus [45].

Total nucleic acid was extracted with the E.Z.N.A. Mag-Bind Viral DNA/RNA Kit (Omega Bio-tek, Norcross, GA, USA). The Vet-MAX Gold BVDV Detection Kit (Applied Biosystems, Foster City, CA, USA) was used for the BVDV qRT-PCR. Cattle samples were pooled in groups of 10. Elk samples were pooled by 5 for seronegative samples. Seropositive samples were processed individually.

#### Herpesvirus

We used the Pourquier® ELISA IBR-IPV Serum gB Blocking kit (Institut Pourquier, Montpellier, France) according to manufacturer instructions on both cattle and elk sera. This test identifies exposure to herpesviruses in general, as there are close genetic and antigenic relationships between BHV1, Cervid Herpesviruses (CerHV), Elk Herpesviruses (ElkHV) and other herpesviruses, particularly within the very conserved glycoprotein B [25,46].

E.Z.N.A. Blood DNA Kit (Omega Biotek Inc., Norcross, GA, USA) was used to extract DNA from the elk serum samples. Seropositive elk samples were processed individually, while seronegative samples were pooled by 5 before extraction. Cattle sera were pooled by 10 before extraction. The polymerase chain reaction (PCR) protocol was modified from Chmielewicz et al. [47], using the primers DFA, ILK, KG1, TGV, IYG. PCR-positive samples were extracted with E.Z.N.A.® Gel Extraction Kit (Omega Bioservices, Norcross, GA, USA) and cloned into a pGEM®-T Easy Vector system (Promega Corporation, Madison, WI, USA) before sequencing (see details in Additional file 1).

#### Mycobacterium avium subsp. paratuberculosis (MAP)

Serum samples from cattle were processed with the IDEXX® *Mycobacterium paratuberculosis* Antibody Test Kit (IDEXX, Westbrook, Maine, USA). To test elk samples, this commercial kit was modified and validated as described in Pruvot et al. [48].

Fecal samples from both species were cultured in our USDA accredited Johne’s disease laboratory using TREK ESP® Culture System (TREK diagnostic systems, Cleveland, OH, USA), as previously described by Forde et al. [49]. MAP culture for the cattle fecal samples was performed in pools of five samples grouped by age and ranch number (150 pools). All samples from positive pools were processed individually [50]. A subset of 30 fecal samples per elk herd were randomly selected and processed individually, allowing for the detection of a prevalence level of at least 10% with 95% confidence. A DNA extraction procedure was performed on the broth of all the fecal cultures before PCR of the target sequence IS*900* as described in [51].

Additionally, DNA was extracted directly from the 86 fecal samples from captured elk, using the MagMAX™ Total Nucleic Acid Isolation Kit (Applied Biosystems, Carlsbad, CA, USA). Negative extraction controls were included in each run. The qPCR protocol^a^ was adapted from [52]. Details of the protocol modifications can be found in the Additional file 1.

#### Neospora caninum

Cattle and elk serum samples were tested in duplicate with a commercial *N. caninum* competitive ELISA kit (*Neospora caninum* Antibody Test Kit, cELISA, VMRD Inc., Pullman, WA, USA) according to the manufacturer’s recommendations and with the kit control samples. ELISA optic density (OD) results were expressed as a percentage of inhibition (%I) of the negative control (%I = Mean OD sample/mean OD negative).

In the absence of *N. caninum*-positive and -negative elk sera, the use of mixture distribution models as described in [53] and a comparison with the VMRD *Neospora caninum* Indirect Immunofluorescence assay kit (VMRD Inc., Pullman, WA, USA) in a Bayesian latent class model (available online [54]) confirmed that the competitive ELISA kit could be applied to elk with the same procedure as described by the manufacturer for cattle (Pruvot, unpublished observations).

### Statistical analyses

Univariate and multivariate analyses were conducted with STATA 11.2 (StataCorp, 2009. *Stata Statistical Software*: *Release 11*. College Station, TX, USA). Fisher exact or Pearson Χ^2^ tests were used to assess the association between species commingling and pathogen occurrence. We used the Wilcoxon-Mann-Whitney (W-M-W) test to compare the values of non-normally distributed variables across groups.

We used multivariate mixed-effect linear or logistic regressions with herd/ranch as random effect (grouping variable) to further test the associations between herpesvirus seropositivity and elk body condition and the associations between inter-species commingling and the occurrences of *N. caninum* in cattle and elk.

## Results

### Ranch characteristics

Two of the ranches without exposure to elk dropped out after the start of the sampling period and could not be replaced. From the 28 remaining ranches, we obtained serum and fecal samples from 848 cows, and 18 additional sera from weak calves from 6 ranches.

To ensure that our sample of cow-calf operations was representative of operations in Alberta, ranch characteristics were compared to provincial averages (Table 2).

**Table 2:** Comparison of surveyed ranch characteristics and provincial average values

	**Surveyed ranch mean (95% confidence interval)**	**Alberta average**^**a**^
Number of wintered cows	174 (135-212)	157
Number of cows per bull	22 (16-28)	26
Conception rate	93% (92-95)^b^	89%
Weaning rate	97% (96-98)	98%
Calf mortality rate	0.9% (0.5-1.2)^c^	2.0%

^a^from [41].

^b^calculated from questionnaire-reported values; 90% (95% CI = 87-92%) if calculated from the subgroup of cows that were checked for pregnancy.

^c^calculated from questionnaire-reported calf mortality over 4 years.

### Risk perception documented by the interview

On a scale from 0 to 4, the average perceived likelihood of disease transmission between cattle and elk was 2.1 (95% CI: 1.2 – 3.0) for non-exposed ranches and 1.5 (95% CI: 0.9 - 2.2) for elk-exposed ranches; this difference was not statistically significant. There was also no significant difference between the two rancher groups in the degree of agreement with the statement “elk disease should be monitored”. Among the 15 ranches exposed to elk, 11 (73%, 95% CI: 48 - 99%) observed occasional direct contact between cattle and elk (as defined by the presence of both species within 2 or 3 meters of one-another); however, in an open question about ranchers’ main concerns regarding the presence of elk on their land, only three cited the possibility of disease transmission. The most commonly reported concern was the consumption of grass on pasture, hay and crops, by 12 of the 15 ranchers, while the second most reported issue was related to damages to fences (by seven ranchers). Eleven out of 15 elk-exposed ranchers took measures to mitigate these concerns, the most common being fencing of haystacks, chasing and hunting. One rancher estimated the annual cost of damages caused by elk up to 10 000 CAD, and a common perception is that ranchers should not bear these costs without compensation.

### Pathogen occurrence in cattle and elk

#### Pestivirus

**Figure 2:**
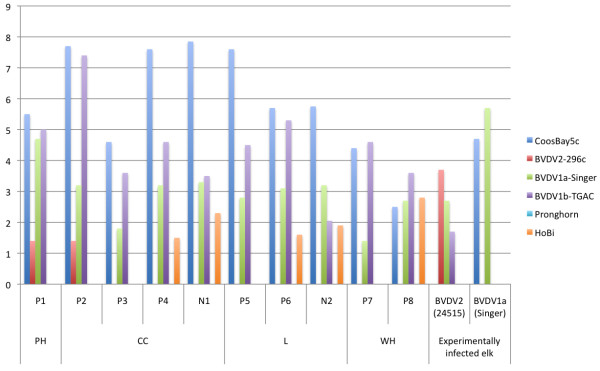
**Virus neutralization test results for 12 elk sera against 6 pestivirus strains.** The y axis indicates the virus neutralization titers in log 2 scale. The x axis indicates the elk sample number: 8 ELISA-positive elk (P1 to P8) and 2 ELISA-negative elk (N1 and N2) captured in herds PH, CC, L and WH; and 2 experimentally infected elk with the BVDV strains BVDV2 24515 and BVDV1a Singer. The second row of the x axis indicate the grouping of the elk samples by origin (herd or experimental infection). For each elk sample, each bar of the graph indicates the neutralization titer for each of the 6 pestivirus strain.

Eight out of 278 elk sera tested positive on the pestivirus antibody ELISA. These 8 individuals were from 4 herds exposed to cattle (CC, L, WH and PH), resulting in a significant effect of the exposure to cattle (Fisher’s exact *p* = 0.002). However, the virus neutralization (VN) test indicated that higher neutralizing titers were obtained for the BDV strain (CoosBay5c) and BVDV1b. When inspected by herd, titers were higher for BDV strains, except for one herd (WH) where both seropositive individuals had marginally higher titers for BVDV1b (Figure 2). In comparison to these ELISA-positive elk, ELISA-negative individuals from the WH and PH herds were negative by VN against all strains, and 2 samples close to the ELISA cutoff were positive in VN and had higher titers against BDV (N1 and N2 in Figure 2). Finally, sera from 4 experimentally infected elk with the strains BVDV1 Singer and BVDV2 24514 had higher VN titers for the homologous strain in two individuals (Figure 2) but high cross-reactivity with BDV strains in 2 other infected elk (data not shown).

None of the elk sera tested positive for pestivirus in the qRT-PCR.

In cattle, 92.6% of all animals were seropositive for antibodies against BVDV (810/875). The seroprevalence was 95.0% (95% CI: 94 – 97%) in vaccinated ranches. In one ranch without routine vaccination strategy against BVDV, 2 cows were seropositive but none of calves tested positive in BVDV qRT-PCR. In vaccinated herds, the proportion of cows with no detectable antibodies ranged from 0 to 23%. BVDV vaccination coverage was significantly lower for individuals below 2 years of age (47%; 95% CI: 23 - 72, Pearson Χ^2^: *p* < 0.001). None of the qRT-PCR for BVDV was positive in cows or weak calves.

#### Herpesvirus

Sixty-four percent (101/277) of the captured elk had evidence of exposure to herpesvirus. Seroprevalence was significantly higher in elk with no cattle contact (71.0 ± 7.5%) compared to exposed elk (55.3 ± 8.6%, Pearson Χ^2^: *p* = 0.007), although this association was only significant for females in 2008 when stratified by year of capture and sex. Females were more likely seropositive than males (*p* = 0.001) and positive animals were significantly older than negative animals (Wilcoxon-Mann-Whitney (W-M-W) test: *p* < 0.001). On a subset of individuals (*n* = 33) for which body weight measurement was available, weights were significantly lower in seropositive animals (235 ± 23 kg) compared to seronegative animals (267 ± 41 kg). This association was still significant (*p* = 0.01) after adjusting for age, sex and girth measurement (used as proxy for body size) in a multivariate linear regression. Four elk samples tested positive by PCR and the sequenced viruses had a close homology (96 to 98%) to a type 2 ruminant rhadinovirus of elk (GenBank: AY237365.1). Elk testing results are summarized by herd in Table 3.

**Table 3:** Test results for four livestock pathogens in elk, by herd

**Herd (abbreviation)**	**Pestivirus**		**Herpesvirus**		**Mycobacterim avium subsp. Paratuberculosis**			**Neospora caninum**
	**cELISA**	**RT-PCR on serum samples**	**cELISA**	**RT-PCR on serum samples**	**Modified ELISA**	**Fecal culture**	**qPCR on MERP fecal samples (captured elk)**	**cELISA**
Beauvais lake (BL)	0/9	0/9	5/9 (*56* ± *34*%)	0/9	0/9	0/30	1/8	1/9 (*11* ± *22*%)
Castle-carbondale (CC)	3/71 (*4* ± *5*%)	0/71	39/71 (*55* ± *12*%)	1/71	1/71 (*1* ± *3*%)	0/30	1/34	3/71 (*4* ± *5*%)
Livingstone (L)	2/15 (*13* ± *18*%)	0/15	10/15 (*67* ± *25*%)	0/15	0/15	0/30	0/12	3/15 (*20* ± *21*%)
Porcupine hills (PH)	1/8 (*13* ± *25*%)	0/8	4/8 (*50* ± *37*%)	1/8	0/8	0/30	0/3	0/8
Whaleback (WH)	2/29 (*7* ± *9*%)	0/29	15/29 (*52* ± *19*%)	0/29	0/29	0/30	1/12	1/29 (*3* ± *7*%)
Waterton (W)	0/16	0/16	10/16 (*63* ± *25*%)	0/16	0/16	0/30	1/10	1/16 (*6* ± *12*%)
Crowsnest pass (CP)	0/17	0/17	11/17 (65 ± 23%)	2/17	0/17	0/30	0/7	0/17
Jasper National Park (JNP)	0/31	0/31	20/31 (*65* ± *17*%)	0/31	1/31 (*3* ± *6*%)	0/30	NA	2/30 (*7* ± *9*%)
Banff National Park (BNP)	0/20	0/20	12/20 (*60* ± *22*%)	0/20	0/20	0/30	NA	0/20
Yaha Tinda (YHT)	0/61	0/61	50/61 (*82* ± *10*%)	0/61	4/77 (*5* ± *5*%)	0/30	NA	2/63 (*3* ± *4*%)

Fractions indicate the *number of positive samples*/*total tested in the herd*, parentheses indicates the *apparent prevalence* ± *standard error*.

Cows had an overall herpesvirus seroprevalence of 98% (95% CI: 97 - 99%) in vaccinated ranches. In 19 cattle herds, 100% of the sampled cows were seropositive, while it ranged from 83 to 97% in the 9 remaining herds. Five ranches did not report any vaccination protocol for calves, and 4 ranches did not have exact knowledge on the vaccination strategy they used for their cows. Seven cows were seropositive cows in one ranch with no routine vaccination strategy, but the PCR tests were all negative for herpesvirus in cows and calves.

#### Mycobacterium avium *subsp.* paratuberculosis *(MAP)*

None of the 386 elk fecal samples collected from free-ranging elk was culture-positive and all culture broth extractions were negative by PCR. From the 86 fecal samples collected from captured animals, 4 fecal samples were positive by direct IS*900*-qPCR. Of 284 elk sera, 4 were ELISA-positive using the original kit cut-off value, while 6 were positive using the newly-defined elk-specific cut-off value [48] (Table 2). There was no association between the presence of beef cattle on the elk home range and the risk of elk being seropositive.

Among the 840 cows tested, 7 were ELISA-positive (95% CI: 0.2-1.4%) from 6 different ranches (21% of participating ranches), 4 exposed and 2 non-exposed to elk. Two cows from the same ranch, unexposed to elk, were fecal culture-positive (confirmed by positive *IS900*-PCR of the extracted culture broth).

#### Neospora caninum

Serology results for *N. caninum* in elk are summarized by herd in Table 3. Elk exposed to cattle had higher percentage of inhibition (%I) than unexposed elk (W-M-W test: *p* < 0.001). In a mixed effect model with the herd and capture year as random effects and adjusted for age and sex, the effect of the exposure to cattle was significantly positively associated with the log-transformed *N. caninum* cELISA%I (*p* = 0.04).

Overall *N. caninum* seroprevalence in cattle adjusted for herd size was 7.1% (95% CI: 5.2-9.1%), while herd-level prevalence (15/28 ranches) was 54% (95% CI: 35 - 72%). In univariate analysis, herds that had no elk on their land had significantly higher prevalence (Pearson Χ^2^ = 4.5, *p* = 0.035), but this association was no longer significant after adjusting for herd clustering in a random-effect logistic regression.

## Discussion

In this study, we determined the presence of production limiting pathogens with various transmission routes in cattle and elk populations from the same geographical area and assessed the association between species commingling and pathogen co-occurrences. To appropriately include the human dimension of this study, we also evaluated the risk perception of inter-species pathogen transmission by the rancher community.

### Risk perception of diseases at the wildlife-livestock interface

Despite the high proportion of elk-exposed ranchers reporting the observation of direct contact between cattle and elk, the perceived risk of inter-species disease transmission was low, and similar between ranches exposed to elk or not. The three ranchers who cited infectious disease transmission as a significant concern associated with elk presence on their land had either previous health issues in their livestock that they attributed to elk, or showed greater overall knowledge on infectious diseases. This may suggest a habituation of ranchers to elk: most of them have been able to cohabit without any major health events that they could attribute to elk and therefore perceive the risk as low. This observation differs from what was described by Brook and McLachlan in a context of bovine tuberculosis transmission between elk and cattle, where the level of risk perception was best predicted by the frequency of elk observation by farmers [55]. This may suggest a shift in the risk perception mechanisms in the context of an immediate threat: the perceived likelihood of inter-species transmission only increases with the intensity of elk presence when a threat is current and clearly identifiable. During the recruitment phase, a few ranchers expressed concerns regarding the consequences our study could have on their activity. One rancher clearly referred to a recent case of a false-positive result in cattle tested for anaplasmosis (resulting in expensive quarantine and testing procedures [56]) and indicated he feared participation in the study. This attitude is also quite prevalent regarding infectious diseases in general, with concerns expressed regarding the legal consequences of detecting pathogens in their livestock (leading some to decline participation). Working with endemic pathogens that do not legally require any reporting or control measure, was a particularly efficient way to decrease this type of concern and ensured a level of trust conducive to successful collaboration with the rural community. Thus, planning studies in a non-emergency context may be particularly beneficial to the advancement of our understanding of multi-host systems.

Perceived risk may not be well correlated to objective risk [57], but is an important factor to consider in study planning, risk communication, and implementation of control measures [58].

### Co-occurrence of production limiting diseases in cattle and elk

Evidence of exposure to the two pathogens with indirect transmission routes, MAP and *N. caninum*, was found in both species. Consistent with previous reports [21], there was a low prevalence of MAP in the sampled cow-calf operations, but we observed high intra-herd prevalence in infected herds. Live bacteria could not be isolated from any of the elk samples, but DNA was detected by qPCR and antibodies by ELISA, suggesting that MAP circulates in some of these wild elk populations. Although the ELISA includes steps to decrease cross-reaction with other Mycobacteria [59] and IS900 is fairly specific for MAP [60], it is still possible that these reactions were due to cross-reacting mycobacteria. Findings for *N. caninum* were also consistent with previous evidence of exposure to this parasite of elk [61] and beef cattle [21].

We showed evidence of pestivirus circulation in elk, and most individuals had the highest neutralizing titers for the BDV strain (CoosBay5c) or BVDV1 strain. However, the difference of titer between strains was not large and could be due to cross-reactivity with a pestivirus strain that was not included in the panel. Serum samples are not optimal for the detection of viral nucleic acid for pestivirus, which may have hindered our ability to detect viremic individuals by qRT-PCR in cattle and elk.

Seropositivity of elk for herpesvirus may be partly due to serological cross-reactivity of elk-specific gammaherpesvirus from the non-MCF (Malignant Catarrhal Fever) subgroup (or type 2 Ruminant Rhadinovirus) [62,63] as indicated by the PCR/sequencing results, or from other elk alphaherpesviruses (e.g. ElkHV1) [25]. Although no clinical disease has ever been reported, the significant lower body weight of infected individuals may suggest subclinical manifestations with possible consequences on elk population dynamics. The observed difference of seroprevalence in females and males might be in part due to differences in herd structure and behavior, where females tend to stay in bigger herds at higher densities, which may increase viral transmission.

The sampling and laboratory procedures did not allow us to assess viral circulation in the cattle herds, in particular due to the cross-reaction between vaccination and serological tests and the limitations in using serum samples for virus detection. However, vaccination is an important ranch management practice altering cattle susceptibility to pathogens, within herd transmission, and consequently the risk of cross-species spillover and spillback. Our survey revealed a high vaccination coverage against BHV1 but significantly lower against BVDV, particularly in young stock, consistent with vaccination coverage in calves entering feedlots (B. Wolfger, personal communication). This may be due to an inadequate vaccination protocol for calves in some ranches, conditions in which it is performed, or interaction of the vaccine strain with maternal antibodies. The level of knowledge on vaccination protocols was highly variable, with many ranchers unable to report confidently their vaccination strategy, which may also contribute to lowering vaccination coverage. Due to the possibility of BVDV-persistently infected (PI) animals shedding large amount of virus for extended period of time, it is recommended that the proportion of vaccinated individuals reach 100% [64]. Furthermore, vaccine-induced antibodies may not always be protective against field strains, it is therefore likely that the level of herd immunity observed in our study still allows BVDV circulation and production of PI calves. The high proportion of immunologically naive calves may increase their risk of infection particularly in periods where these calves are exposed to other susceptible animal species infected with pestiviruses or other cattle herds, for example during summer grazing on public land or community pasture.

Sample collection in wildlife presents some challenges due to the absence of exact census data, the inability to identify individuals and, for wild cervids, the distribution of individuals into unstable sub-groups moving across a large territory. Our application of a systematic sampling strategy for fecal sample collection overcame some of the challenges associated with non-random sampling of wildlife populations [65] and ensured the collection of fresh samples from distinct individuals in nine elk herds. Some more remotely located sub-groups may not have been sampled, which may bias our sample toward the most visible resident herds, but conversely provides better knowledge of their level of exposure to cattle.

Similarly, with elk serum samples collected through the various projects, a word of caution is necessary regarding inferences in these elk populations, as protocol variations between projects and year-to-year variations in sample selection (in adaptation to primary objectives of these projects) may have biased our sample. In particular, male elk were under-represented in the samples and were often young, thereby possibly underestimating prevalence in this sex group.

### Effect of inter-species commingling on pathogen occurrence

An interesting result from our study was the apparent effect of cattle on the prevalence of *N. caninum* in elk, as elk in contact with cattle had a higher risk of being seropositive. Conversely, the effect of elk exposure on the occurrence of *N. caninum* in cattle was not significant in multivariate analysis. This may indicate a predominant vertical transmission in beef cattle (from dam to calf), therefore little influenced by the density of susceptible host, while horizontal transmission between cattle, carnivores and elk may increase the risk for elk in areas used by infected cattle [66]. The distribution of MAP in elk was not associated with their exposure to cattle, which may suggest circulation in elk independently of contact with cattle. This has been previously reported in wild [29] and farmed [31] elk. However, the absence of statistical effect of species commingling on MAP occurrence does not rule the possibility of MAP inter-species transmission out, and a previous study in Norway found an OR = 4 (3-5) for the presence of red deer on cattle pasture in a MAP case-control study in dairy herds [67]. Finally, regarding the directly transmitted viruses: gammaherpesvirus found in elk were unrelated to cattle BHV1; and only elk in contact with cattle had evidence of pestivirus circulation although these pestiviruses could not be definitely ascertained to be of livestock origin.

For the purpose of this manuscript, elk herds and ranches were assumed to be independent and their exposure to be constant and broadly defined as a binary variable to constitute two groups. Despite the uncertainty related to elk winter survey data and telemetry data from a limited number of collared elk used for the classification, and some seasonal and year-to-year variability, these patterns can reasonably be assumed to be stable enough to define a long-term steady exposure for each ranch and elk herd. However, some elk herds have overlapping home ranges, exchange dispersal individuals and adapt their movement based on environmental and climatic conditions over time which challenges a simple dichotomous measure of exposure and most classical statistical approaches. Also, both cow-calf operations and elk herds have particular habitat preferences: there may therefore be a number of other environmental factors confounding the relationship between interspecies contact and disease occurrence.

The ranch selection was also subject to biases due to the initial method of recruitment and thereafter, ranchers’ interest in the study and acceptance to participate; no cow-calf operation census listing was available due to privacy protection reasons. Although the ranch characteristics were consistent with previous Alberta cow-calf operation benchmark data [41], interpretations and generalization should be made cautiously, particularly regarding aspects of risk perception.

### The importance of the transmission route

Consistent with the hypothesis that pathogens with indirect transmission routes may be more likely shared in a multi-species system, we found *N. caninum* to be more prevalent in elk in contact with cattle, and evidence of MAP circulation in elk; whereas evidence of pathogen sharing for directly transmitted herpesviruses and pestiviruses was equivocal. While a tight spatio-temporal overlap at defined periods would be required for the direct (or close indirect) transmission of BVDV and BHV1 (possibly facilitated by winter feeding, summer grazing in forestry areas, hay stacks, mineral blocks, water sources, natural licks, open feeders), the long persistence of MAP in the environment [68,69] may relax the temporal constraints. The transmission of *N. caninum* involves more complex ecological processes due to the existence of vertical and horizontal transmission, and domestic and sylvatic cycles. When horizontal transmission is predominant, the spatio-temporal proximity may not be as critical as for directly transmitted pathogens, due to the mobility of wild and domestic carnivores and the persistence of the infection in these definitive hosts.

Our selection of endemic livestock diseases did not fully allow controlling for the pathogen intrinsic factors influencing the potential for inter-species transmission, in particular due to the difference of host susceptibility for herpesviruses and pestiviruses between cattle and elk. Cattle vaccination has also limited our ability to assess intra and inter-species virus circulation. The low prevalence and slow development of MAP infection may also be shortcomings for MAP as a model for environmental transmission. Highly prevalent and environmentally persistent pathogens such as *Cryptosporidium sp*., *Giardia sp*. or *Escherichia coli*[70] may be interesting models to consider for future research. Additional qualitative and quantitative measurements of cattle/elk interactions may also help to further disentangle intrinsic and extrinsic factors linking transmission route and inter-species transmission. With the spatio-temporal contact structure between species clearly identified, transmission routes may be an efficient way to locally prioritize pathogen surveillance in a multi-species grazing system. A clear understanding of the perceptions and attitudes of the different stakeholders toward wildlife, livestock and the risk of inter-species pathogen transmission, is essential to ensure effective communication and successful collaboration.

## Endnotes

^a^Our laboratory has received certification through the USDA National Veterinary Services Laboratories (NVSL) Johne’s disease direct PCR proficiency test.

## Competing interests

The authors declare that they have no competing interests.

## Authors’ contributions

MP contributed in conceiving and designing the study, collected cattle samples and elk fecal samples, participated in collecting captured elk samples, conducted interviews, carried out part of the laboratory analyses, analyzed data and drafted the manuscript; SK contributed to conceiving and designing the study and drafting the manuscript, FVDM contributed to the laboratory analyses of cattle and elk samples and contributed to drafting the manuscript; MM contributed to conceiving and designing the study, collecting captured elk samples and drafting the manuscript; HWB contributed in designing the project and drafting the manuscript; KO contributed in conceiving, designing and coordinating the project, participated in the data analysis and the manuscript drafting. All authors read and approved the final manuscript.

## Supplementary Material

Additional file 1 **Supplementary laboratory protocol information.** This document provides additional information about laboratory procedures for the MAP qPCR and Herpesvirus PCR carried out on fecal and serum samples respectively. Click here for file
