# Supplementary material for: Pathogens at the livestock-wildlife interface in Western Alberta: does transmission route matter?
Source: Vet Res. 2014 Feb 12;45(1):18. doi: 10.1186/1297-9716-45-18 (PMC3937035; doi:10.1186/1297-9716-45-18)
Supplement: Additional file 1 — Supplementary laboratory protocol information. This document provides additional information about laboratory procedures for the MAP qPCR and Herpesvirus PCR carried out on fecal and serum samples respectively. [file 1297-9716-45-18-S1.doc]

**Supplementary laboratory protocol information**

**Herpesvirus PCR**

The first round PCR mix (10 µL D Buffer, 1 µL DFA primer, 1 µL ILK primer, 3.8 µL water, 0.2 µL Taq polymerase, 3 µL template) was heated for 5 min at 95 °C before to run through 45 cycles (95 °C – 30 s; 46 °C – 60 s; 72 °C – 90 s) and a final termination step of 5 min at 72 °C. The second round PCR mix (10 µL F or H or D Buffer, 1 µL TGV primer, 1 µL IYG primer, 5.8 µL water, 0.2 µL Taq polymerase, 2 µL template) followed the same cycling protocol except that the step 2 and 3 of the cycles were 30 s and 60 s respectively.

**MAP qPCR**

During the bead beating step of the MagMAX^TM^ Total Nucleic Acid Isolation Kit (Applied Biosystems, Carlsbad, CA, USA) procedure, samples were beaten twice for 5 min on a Mini BeadbeaterTM (Biospec, Bartlesville, OK, USA), and cooled on ice between beatings. Samples were processed in the 96-well plate provided with the MagMAX kit, using a 96 well Magnetic-Ring Stand (Applied Biosystems) for bead capture. DNA was eluted in 50 µL of the provided elution buffer heated to 56 °C.

Cycling conditions were: 50 °C for 2 min, 95 °C for 20 s, 40 cycles of 95 °C for 3 s and 61 °C for 30 s, 95 °C for 1 min and extension for 72 °C for 5 min. Primer and probe concentrations were also optimized to 10 pmol of both forward and reverse primers, 1pmol of both probes, approx. 900 copies of internal control plasmid. We used the TaqMan® Fast Advanced Master Mix (Applied Biosystems).
